# Impact of Climate Change on Crops Adaptation and Strategies to Tackle Its Outcome: A Review

**DOI:** 10.3390/plants8020034

**Published:** 2019-01-30

**Authors:** Ali Raza, Ali Razzaq, Sundas Saher Mehmood, Xiling Zou, Xuekun Zhang, Yan Lv, Jinsong Xu

**Affiliations:** 1 Key Laboratory of Biology and Genetic Improvement of Oil Crops, Oil Crops Research Institute, Chinese Academy of Agricultural Sciences (CAAS), Wuhan 430062, China; snookas.saher90@gmail.com (S.S.M.); lvyan01@caas.cn (Y.L.); xujingsong@caas.cn (J.X.); 2 Centre of Agricultural Biochemistry and Biotechnology (CABB), University of Agriculture, Faisalabad 38040, Pakistan; ali.razzaq254@gmail.com

**Keywords:** crop adaptation, climate change, genetic engineering, genome wide association studies (GWAS), marker-assisted selection (MSA), molecular breeding, hormone responses, physiological responses

## Abstract

Agriculture and climate change are internally correlated with each other in various aspects, as climate change is the main cause of biotic and abiotic stresses, which have adverse effects on the agriculture of a region. The land and its agriculture are being affected by climate changes in different ways, e.g., variations in annual rainfall, average temperature, heat waves, modifications in weeds, pests or microbes, global change of atmospheric CO_2_ or ozone level, and fluctuations in sea level. The threat of varying global climate has greatly driven the attention of scientists, as these variations are imparting negative impact on global crop production and compromising food security worldwide. According to some predicted reports, agriculture is considered the most endangered activity adversely affected by climate changes. To date, food security and ecosystem resilience are the most concerning subjects worldwide. Climate-smart agriculture is the only way to lower the negative impact of climate variations on crop adaptation, before it might affect global crop production drastically. In this review paper, we summarize the causes of climate change, stresses produced due to climate change, impacts on crops, modern breeding technologies, and biotechnological strategies to cope with climate change, in order to develop climate resilient crops. Revolutions in genetic engineering techniques can also aid in overcoming food security issues against extreme environmental conditions, by producing transgenic plants.

## 1. Introduction

Natural systems, human health, and agricultural production have been badly affected by devastating environmental changes [1]. With the rapid increase in the world’s population, there is a corresponding increase in food demand owing to concerns about the stability of the global environment. Water availability, air pollution, and soil fertility have a large impact on agriculture productivity [2]. With abrupt changes in environmental conditions, the harsh impacts on plant productivity are progressing in great intensities owing to direct and indirect effects of abiotic stresses. Because of the continuous deforestation and excessive utilization of fossil fuels, the concentration of CO_2_ has escalated from 280 µmol^−1^ to 400 µmol^−1^ in the atmosphere. It is predicted that the CO_2_ concentration will elevate two-fold, i.e., up to 800 µmol^−1^ at the end of this century. Emission of dangerous gases, especially CO_2,_ are the main factors for the greenhouse effect and warmer average global temperatures [3]. The effects of climate change and environmental variation are mainly estimated by the number of stress spells, their impact on daily life, and damage to agricultural crops [4]. In developing countries, agricultural yield is predominantly suffered due to adverse environmental conditions, therefore high temperature and excess of CO_2_ accumulation forced scientists to devise new strategies to cope with less predictable challenges [5]. To tackle these limitations and guaranteed food security there is a need for production of new climate-smart crop cultivars [6]. Plant growth and yield are greatly influenced by abiotic stresses. Under natural climate conditions, plants often experience numerous stresses like waterlogging, drought, heat, cold, and salinity [7,8]. The abiotic factors also include UV-B, light intensities, flooding, gas emissions, and physical and chemical factors which induce more stresses [9]. In the 21st century, the Earth’s average temperature is expected to increase from 2 to 4.5 °C. According to IPCC-2014 (http://www.ipcc.ch/), the time-span between the 19th and the 21st centuries is considered to be the period which experienced the most warming [10]. Extreme precipitation events might well cause destructions due to floods whereas the scarcity or the total absence of rainfall for a longer period of time leads to drought stresses [11]. The environment of the globe is continuously changing and industrialization is one of the main factors for temperature increase. Due to extreme weather events the frequency of global warming is expected to rise, which will ultimately disturb the ecosystem globally [12]. All living organisms such as plants, animals, fishes, and humans have been affected by the extreme environmental conditions around the globe. The danger to the world’s climate conditions has triggered anxiety among everyone because crop yield might be compromised by fluctuations in various environmental factors that can risk food security. Recent studies reported that the developed countries have more vulnerability towards climatic changes (8–11%) than developing states [13,14]. Climate change and food insecurity are the two major issues of the 21st century. Around 815 million people are affected by malnutrition, hindering sustainable development programs to achieve the universal goal of eliminating hunger by 2030 [15]. Food security and agricultural yield is considerably affected by the adverse weather. With elevation in temperature, the production of major crops has been reduced evidently around the world [16]. At the end of this century global production of crops is likely to decrease as climatic severity increases from 2.6 to 4 °C. Reduction in the productivity of these crops signifies the main threat to food security, particularly in the speedy increase in the world’s population [17]. The population is supposed to grow to about 9 billion in 2050 and food requirement are expected to escalate by about 85% [18]. Climatic influences are worsened by present cropping schemes with low variation and elevated concentration of inputs, and unstable productivity due to environmental changes in crops [19]. The increased frequency of drought and heavy rainfalls, temperature fluctuations, salinity, and insect pest attacks are anticipated to decrease crop productivity leading to higher threats of starvation [20]. Crop adaptability has suffered not only as a result of temperature variations, but also because of rainfall [21]. Currently, the main task is lessening the pressure on food security [22]. This review emphasizes the influence of weather variations on crop production. The next sections outline the overview of the climate change, stresses produced due to climate change, impacts on agricultural crops, strategies to cope with extreme environmental conditions, and some recent genetically engineered approaches to develop transgenic plants against abiotic stresses. 

## 2. Plant Yield and Climate Change

**Figure 1:**
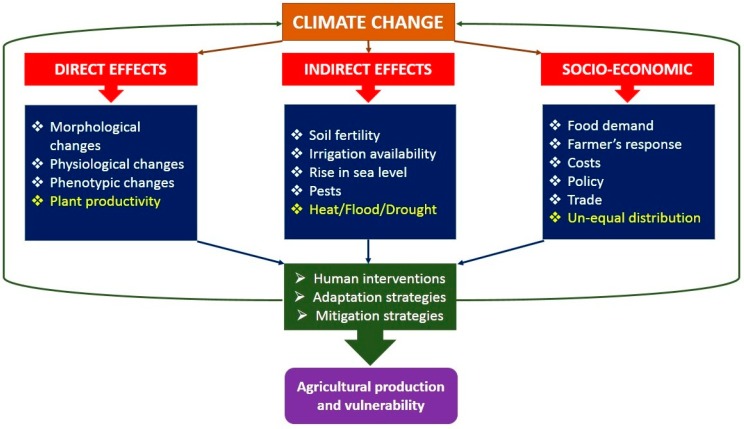
Direct, indirect and socio-economic effects of climate change on agricultural production.

**Figure 2:**
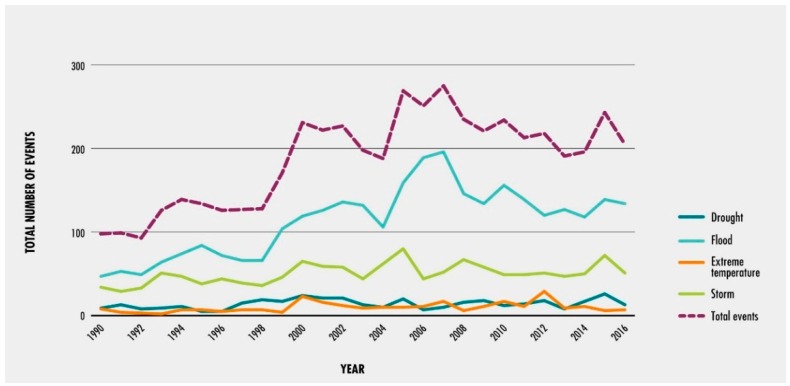
Increasing number of extreme climate-related events occurred during 1990–2016. Source: Food and Agriculture Organization (FAO) based on data from Emergency Events Database (EM-DAT) (https://www.emdat.be/) [24,25].

Plant physiology has been greatly influenced by climate variability by several means. Environmental extremes and climate variability enhanced the chances of numerous stresses on plants [23]. Climate change affects crop production by means of direct, indirect, and socio-economic effects as described in Figure 1. Furthermore, climate change (drought, flood, high temperature, storm etc.) events are increased dramatically as reported by Food and Agriculture Organization (FAO) and as shown in Figure 2.

Boyer reported that the climate changes have reduced the crop yield up to 70% since 1982 [26]. According to the study of FAO 2007 (http://www.fao.org/home/en/), all cultivated areas in the world are affected by climatic changes and only 3.5% of areas are safe from environmental limitations (for detail look table 3.7 in http://www.fao.org/docrep/010/a1075e/a1075e00.htm) [27]. Whereas the outcomes of abiotic stresses on crop yield are hard to calculate accurately, it is believed that abiotic stresses have a substantial influence on crop production depending upon the extent of damage to the total area under cultivation. In future, the productivity of the major crops is estimated to drop in many countries of the world due to global warming, water shortage, and other environmental impacts [28,29].

Based on national crop yields and questionnaire surveys, large differences in vulnerabilities to current climate changes were detected across Europe. In Northern Europe, the short duration for crop development and cool temperature are the major concerns, while the temperature extremes and low rainfall limits the crop productivity in Southern Europe, although the most negative effects will be found for the continental climate in the Pannonian zone, which includes Hungary, Serbia, Bulgaria, and Romania [30]. It was predicted that the enhancement of greenhouse gas emissions and abrupt climatic changes will occur that may increase the crop yield in North-Western Europe and decrease the crop yield in the Mediterranean area [31]. Wheat production is heavily affected by the temperature extremes due to climate change in many countries, and may reduce the crop yield by 6% for each °C rise in temperature [32]. Drought and high temperatures are key stress factors with high impact on cereal yields [33], and *Rubisco*, the central enzyme of photosynthesis, is disrupted if the temperature increases from 35 °C, and stops the photosynthetic process [34]. Gong et al. (1997) reported the negative influence of heat stress on antioxidant enzymes in *Zea mays* [35]. The combined impact of heat and drought stresses on crop yield have been examined in sorghum, maize, and barley. It was revealed that the combined effect of heat and drought stress had more damaging outcomes as compared to individual stress [36]. Xu and Zhou (2006) subjected the *Leymus Chinensis* under the combined stresses of drought and heat and found that the function of Photosystem II (PSII) decreased [37]. 

Due to climate change, water deficit and temperature extremes influence the reproductive phase of plant growth. It was described that the flower initiation and inflorescence is badly affected by the water stress in cereals [38]. Similarly, if the temperature increase of about 30 °C during floret development it can cause sterility in cereals [39]. During the meiotic phase, wheat and rice suffered from the 35–75% reduction in grain set due to water deficit [40,41]. In rice, drought stress greatly disturbs the process of fertilization and anthesis. Due to water deficit, the harvest index is reduced to 60% and decreases the grain set [42]. The cocoa yield has been significantly reduced by the major drought spells in West Africa during the 1980s El Niño years [43]. It has been estimated that agricultural production could reduce to 25.7% by 2080 due to climate change and maize will be the most affected crop in Mexico [44]. A study based on ECHAM6 climate data was analyzed for North German Plains during two different time durations: 1981–2010 and 2041–2070. The results showed that if the yield for winter wheat is to be sustained, water availability must be guaranteed [45]. Zhao et al (2017) carried an experiment to analyze the climate change impact on major crop yields and showed considerable yield reductions of 6%, 3.2%, 3.1%, and 7.4% in wheat, rice, soybean, and maize respectively [46]. To tackle the climate change new discoveries in genomics are enabling climate-smart agriculture by developing climate resilient crops [47].

Drought stress influences wheat during all developmental stages, but grain formation and the reproductive stage are the most critical ones [48]. Wheat yield was decreased from 1% to 30% during the mild drought stress at post-anthesis while this reduction increased up to 92% in case of prolonged mild drought stress at flowering and grain formation [49,50]. Drought stress has greatly reduced the yield of important grain legumes. Mashbean (*Vigna mungo* L.) yield has been reduced by drought stress from 31% to 57% during the flowering stage while a 26% reduction was reported by drought stress during the reproductive phase [51]. Maleki et al. (2013) reported that the soybean yield has been largely effected by drought stress and a 42% reduction was observed during the grain filling stage of soybean [52]. Schlenker and Roberts (2009) described that maize yield was increased at an optimum temperature of 29 °C but a further increase in temperature hampered the yield of maize [53]. Every 1 °C rise in temperature was found to negatively influence the maize yield [54]. Similarly, it was reported that yield in maize decreased by 8.3% with every 1 °C rise in temperature from the optimum growth temperature [55]. Brown (2009) reported that wheat yield decreased by 10% with every 1 °C increase in temperature [56]. In another report it was revealed that a 3–4% reduction in wheat yield takes place for every 1 °C increase in temperature [57]. Easterling et al. (2007) described that a 2 °C increase in temperature cause 7% reduction in yield while a further increase in temperature to 4 °C decreased the yield by up to 34% in wheat. Similarly, rice yield decreased by 2.6% for every 1 °C rise in temperature [58]. In sorghum, yield was reduced by 7.8% due to a 1 °C increase in temperature [59]. In sorghum, water shortage is another big issue reported in most of the world’s top producer countries [60]. Schlenker and Roberts (2009) revealed that the threshold temperature for soybean is 30 °C; a rise in temperature to the optimum level increased soybean yield but after that level, further rise in temperature reduced the yield abruptly [53]. Eastburn et al. (2010) reported that the rise in ozone and CO_2_ concentration in the atmosphere influenced the disease type, and with a continuous rise in temperature, disease susceptibility in soybean was enhanced [61].

**Figure 3:**
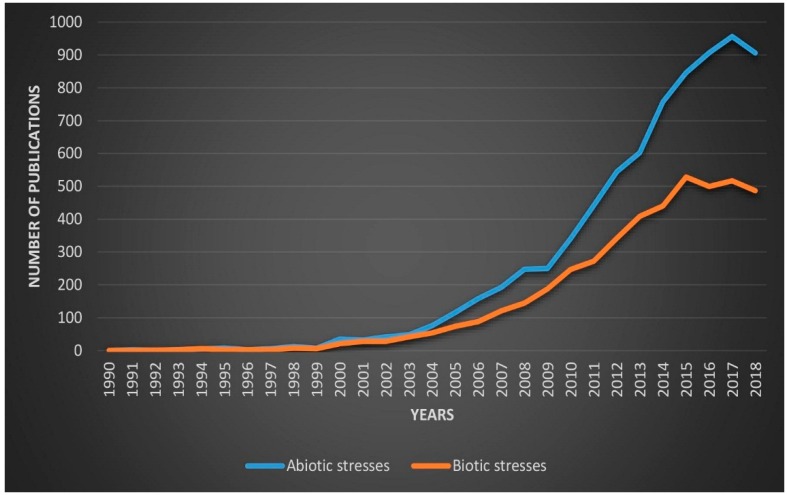
The number of publications per year related to abiotic and biotic stresses from Jan/1990–Nov/2018. Source: PubMed (Keywords (abiotic stresses, drought, cold, heat, salinity and water-logging), (biotic stresses, bacteria, virus, fungi, insects, parasites, and weeds) used to search the number of publications in PubMed).

This rising concern was revealed in the growing quantity of research papers focused on abiotic problems after the crucial review by Kitano on systems biology [62]. The amount of research studies has increased dramatically related to biotic and abiotic stresses in plants by applying different strategies (Figure 3).

Climate change influences food security in a very complicated manner. It hampers the agricultural yield directly by means of disturbing the agro-ecological environment and indirectly by putting pressure on growth and circulation of income and consequently increased the necessity of agricultural products. Impacts of climate change on food security have been calculated in several ways [63]. Here we briefly discuss the potential impact of climate change and food security. 

In temperate regions and humid grassland zones, a slight elevation in temperature may raise the pasture productivity. These advances have to be established to tackle amplified rate of climate changes, for example, drought and temperature extremes in the Mediterranean zone or massive rainfall spells and in temperate areas increase the risk of flooding [64]. But in the case of arid and semiarid regions, it may cause a reduction in livestock growth and enhance their death rates [65]. The extensive rate of evapotranspiration and less moisture in the soil are predicted in drier regions by various climate models [63]. Consequently, due to climate changes, many regions of cultivated land may become unsuitable for cultivation, and other tropical regions may produce more crops. Temperature instability will also provide more favorable environmental conditions for insect pests of crops to boost their capacity to stay alive in cold temperatures and then emerge in outbreaks in spring. It is very crucial to observe that in case of food accessibility, all recent calculations for food security and safety have concentrated mainly on the effects of climate changes in ways that did not measure the probability of substantial alteration in the rate of climate extremes on crop productivity. They have also not considered the situations of sudden changes in socio-economic status and climate, so all these factors have been putting negative impacts on global food security and safety [64]. Around the globe, food security is remarkably significant for human beings. Because of climate change, food quality, supply, and safety are still the biggest problems for researchers. Future studies on food security will need to incorporate climate change, crop productivity, water supply, and population to estimate food security conditions entirely and scientifically [21].

## 3. Crop Adaptation to Overall Extreme Climate Stresses

**Figure 4:**
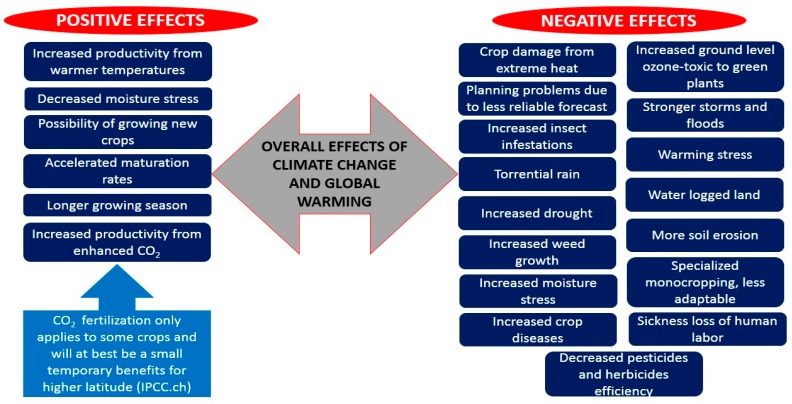
Overall positive and negative effects of climate change and global warming on crops and humans.

With the increase of the Earth’s temperature, the climate undergoes severe alterations and becomes abiotically stressful. Environmental changes are very damaging and pose various threats to naturally prevailing crop species [66]. Under field circumstances, drought and heat are the most predominant stresses and have a significant influence on plants [67]. It is reported that plants require an optimum temperature for their normal growth and blooming. Plant physiology is heavily influenced by temperature fluctuations [68]. As heat stress affects the grain production and yield, cold stress results in sterility, and drought stress negatively influences the morpho-physiology of plants [69,70]. These climatic problems severely distress plant development and yield, produce enormous responses, comprising molecular, biochemical, physiological, and morphological modifications [71]. Overall, global warming and climate change both have some negative and positive effects on agricultural crops as well as on humans as explained in Figure 4.

In this context, understanding the stress-resistance processes in plants has emerged as a very difficult task for plant scientists in order to develop stress-resistant plants [72]. The chief cereal crops around the world, such as maize, rice, and wheat, are crucial to meet the daily food demand. Out of them, wheat was the leading staple crop which has been cultivated on a large scale [73]. Wheat is harvested on 38.8% of total agricultural land worldwide and provides a considerably high concentration of proteins: 15% per gram as compared to maize or rice which only supplies 2 to 3% [74]. Regardless of large growing land globally, its productivity has been predominantly less than the maize and rice [18]. Reasonable reduction was anticipated in wheat productivity with a 2 °C increase in temperature. Related research on environmental variability expected a 6% reduction in wheat yield [75]. Challinor and his colleagues described that due to the increase in temperature, the grain filling phase decrease is the major reason of crop productivity reduction in changing climatic conditions [76]. Therefore, sustaining crop yield is an important task in current agriculture, and to produce stress-tolerant crop plants [75].

## 4. Various Limiting Factors for Crop Development

For sustainable agriculture and food safety for an increasing population of the world, it is necessary to grow stress-tolerant plants and understand their responses under different stress conditions. In relation to various climatic stresses, the response of plants varies in the expression of genes, physiology, and metabolism. It was reported that plants have the ability to sense any variation in surrounding environmental signals but in spite of many studies, only some reputed sensors have been recognized [77]. Due to different stresses, the organs and tissues of the plants are damaged and they respond accordingly, for example, transcriptional responses against various stresses are different in specific cells or tissues of roots [78]. Stress-responsive protein creation, high levels of associated solutes, and more elevated antioxidant ratios are the cellular signals which are produced due to salinity, drought, and chemical effluence. These stresses are regarded as primary stresses and they generate secondary stresses like oxidative and osmotic stress [79].

Under drought conditions, elevated level of CO_2_ in leaf causes the initiation of reactive oxygen species (ROS) which trigger the multiple stresses in crops. With locked stomata, movement of CO_2_ inside the leaf is clogged, and ROS are produced due to enhanced levels of oxygen under drought conditions. The frequency of plant development, photosynthesis, and respiration are disturbed by membrane breakdown due to ROS production. Several cell building materials like carbohydrates, lipids, proteins, and nucleic acid are impaired by ROS in drought stress [80]. In recent studies, it was observed that Osmo-protectants have been produced under the combined stress conditions of heat and salinity in tomato plants, but do not appear in individual stresses. Another experiment demonstrated that the combined effect of heat and salt stress leads to diverse metabolomic profiling which was established with molecular and physiological statistics. For plant development, ROS has a significant role and it is considered as a crucial secondary signal for cellular metabolism: an elevated level of ROS prompts cell apoptosis. Therefore, a gentle equilibrium among ROS creation and their decontamination may occur in every oxygenated organism [81]. The adaptability of *Arabidopsis* to persistent water deficiency at the molecular and morpho-physiological levels was examined. *Arabidopsis* collected from various habitations presented alterations at the transcriptomic level [82].

Metabolic profiling of various crucial plants have been comprehensively completed under water stress, such as rice, soybean, maize, and tomato. In barley, numerous metabolomic analyses have also been conducted to understand the impact of water scarcity on the oxidative phase, abscisic acid, and free amino acids. Barley cultivars were subjected to water shortage to explore the genetic variation on the metabolomic level at grain formation phase [83]. Protein production inhibition is the initial metabolic signal against the abiotic factors [84]. Post-translational modifications and processing are also the primary responsibilities of abiotic stresses [85]. Drought stress in coffee has been studied from a wide viewpoint by assimilating the vital features of plant biochemistry and physiology. The plants subjected to multiple events of constant drought stresses have greater photosynthesis processes, in contrast to plants with only one event of drought stress imposed on them. Certainly, these plants showed advance *RuBisCo* control and several enzymes related to metabolism. Adaptability to various drought doses elaborated the gene expressions associated with drought resistance [86].

## 5. Impact on Plants’ Morpho-Biochemical and Physiological Processes

With great environmental variability, plants are suffering from unique climatic conditions that limit the plants’ ability to adapt successfully in a range of ways. Due to more spells of rainfall and warmth, plant relocation is not to be the solution to this problem. However, modifications in plant physiology have been beneficial in unique climatic conditions, but environmental variability can be risky for plants [87]. Morphological, biological, and biochemical mechanisms of plants have been severely affected by abiotic stresses. Although for expected weather conditions in the future, plant physiology reactions are predicted to propagate quickly, with minor variation in fruiting and flowering [88,89]. The ideal temperature for plant development is in the range of 10 to 35 °С. Elevation of the temperature to a specific point will permit plants to generate excess energy but a larger increase in temperature retards the plant growth and the photosynthesis rate abates to deadly levels [90].

Turgor pressure is limited by the drought stress and therefore delays cell development. Water shortage impacts the photosynthesis enzymes actions and decreases the metabolic competency and ultimately destroys photosynthetic machinery [91]. Because of environmental changes CO_2_ levels proliferate and retard respiration in plants and enhance temperature level. Respiration rates of the plant were elevated when the temperature was raised from 15 to 40 °C, disturbing morphological features of some crops [92,93]. During the process of photosynthesis, the enzyme *Rubisco* is associated with carbon fixation and translation of CO_2_ into a complex energy-rich compound [94]. *Rubisco* is activated by the *Rubisco* activase at an optimum temperature by abolishing secondary metabolites. A minor elevation in temperature resulted in the deactivation of *Rubisco* enzyme leading to the generation of xylulose-1,5-bisphosphate which is supposed to be an inhibitory compound. At an increased temperature, *Rubisco* did not work properly because of the Rubisco activase breakdown and was unable to activate *Rubisco* [95]. ROS containing OH, H_2_O_2_, and singlet oxygen are derivatives of metabolisms and are regulated by antioxidant defense mechanism. ROS are mostly formed in minimum amount under optimum conditions but with the increase in concentration environmental stress triggered [96].

## 6. Plant Hormone Responses in Abiotic Stresses

**Figure 5:**
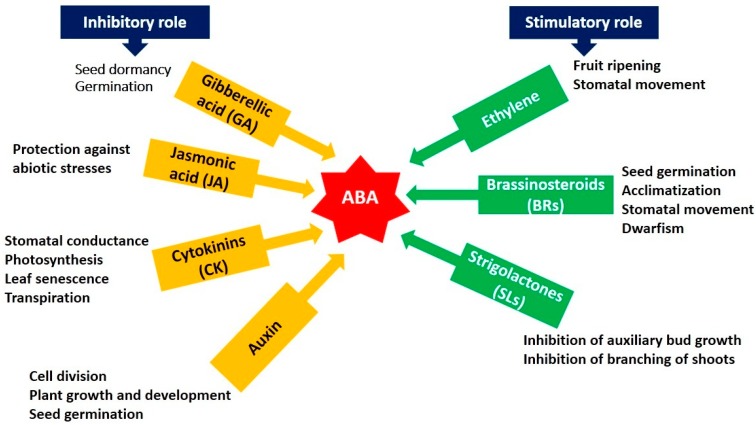
Hormonal crosstalk related to different stresses.

Under different abiotic stresses, hormones are very crucial for regulating many signaling pathways and responses such as salicylic acid (SA), abscisic acid (ABA), and ethylene [97]. The major role is played by ABA in the regulation of stress responses by the interactions with some other hormones as shown in crosstalk (Figure 5). The most important and vital hormone for regulating the climatic stresses in the plant is ABA. ABA plays a major role in different stages of plant development particularly in stomata opening and closing, drought stress, seed germination, and dormancy. PYR/PYL/RCAR-PP2C-*SnRK2* is recognized as a signaling cascade generated by ABA and controls the seed dormancy efficiently. Under drought conditions, the plant growth is severely retarded and it increases the ABA concentration in cells. ABA accumulation during drought stress controls transpiration and inhibit stomatal disclosure [98]. ABA also triggers many physiological mechanisms in plants such as water scarcity, regulates stomata to close down, and produces many stress-responsive genes in this period [99]. ABA signaling machinery have been investigated recently and their mechanism of operation was elucidated. The signaling cascade consisted of 3 units, SnRK2/OST1 (Protein kinase), PP2C (protein phosphatases) and PYR/PYL/RCAR proteins [100].

Two different group of scientists found the ABA PYR/PYL/RCAR receptors [101,102]. PP2C was first observed in *Arabidopsis* knockouts of abi1-1 and abi2-1 and is regarded as the negative controller of ABA [103]. Similarly, the protein kinase was collected and separated as *SnRK2* and it is the activator of ABA [104]. Salicylic acid also has regulated numerous physiological processes in plants under stress climatic condition. It was identified that acetylsalicylic acid can encourage protoplast cluster development in corn, controlling cell cycle regulation [105]. The role of SA was discovered by a group of scientists working on cell cultures of tobacco, they found that SA regulates the bud development and flowering initiation [106]. Malamy and his colleagues were pioneers in studying the Tobacco Mosaic Virus and established the part of SA in plant-pathogen interaction [107]. Recent studies on SA described its impacts on fruit productivity, legumes nodulation, temperature resistance, stomata closing, respiration, genes related to senescence, and cell growth [107]. Salicylic acid regulations in these events might be secondary because they control the production of further plant stress-responsive hormones [108,109]. 

Phytohormones play a vital part in stress response by modulating different signal transduction mechanisms under climatic variability. One of the most important members of phytohormones is ethylene. It is found in gaseous form and thus enables plant-to-plant connections. A century ago, ethylene was discovered and since then many research studies were carried out to reveal its biosynthesis. Ethylene has a function in the control of seed germination, ripening, leaf growth, and senescence under different abiotic and biotic climatic stresses. It is supposed that ethylene acts as signaling pathway among plant growth and weather variations. Abiotic stresses such as salinity, water logging, high temperature, frost, heavy metal contact, nutrient deficiency, and drought are the reasons which modulate the synthesis of ethylene [110]. Ethylene response factors (ERFs) in plant ethylene belong to a massive transcription factors (TFs) family and are activated during the different physiological and environmental stresses. There have been extensive investigations on ERF proposing its role in abiotic stresses but until now there is no evidence of any specific signaling pathway under abiotic stresses. In a recent study, ERFs in tomato were subjected to drought, salinity, heat, cold, and excessive water conditions for their expression profiling [111].

## 7. Approaches to Combat Climate Changes

Variation in the environment has a long-lasting influence on agriculture and food security globally. Food security and safety are threatened by the severe weather conditions and it is not a recent problem. But formerly, no consideration was adopted to tackle this problem. Therefore, to cope with these weather variations is the most urgent demand worldwide. For crops to adapt to changing environmental stresses subsequent approaches are required. 

### 7.1. Cultural Methodologies

Recently some experiments reported investigations of the strategies trialled by farmers to tackle the climatic variation for plant adaptation. There are many useful approaches adopted by farmers, including abiotic factors such as altering planting and harvesting time, a collection of crops with short life cycles, crop rotation, irrigation techniques, and variation in cropping schemes. Under climatic stress conditions, all of these approaches are very beneficial for crop adaptability [112,113,114,115]. Modification in sowing time, application of drought resistant cultivars, and the cultivation of new crops are some important strategies to lessen the climatic variability danger and provide better adaptability to crop plants for assuring food safety and security [116]. Another plant adaptability approach is by means of crop-management techniques that have the ability to enhance crop development under various environmental stresses. The choice of sowing time, planting density, and optimum irrigation practices are crucial techniques to tackle weather stresses [117]. Fertilizers are also very vital to reduce the effect of global warming and support the plant for better adaptability. It provides substantial energy to plants and is beneficial to maintain the fertility of the soil and increase productivity. Hence, the importance of fertilizer in nourishing the world is undeniable [118]. 

### 7.2. Conventional Techniques

**Figure 6:**
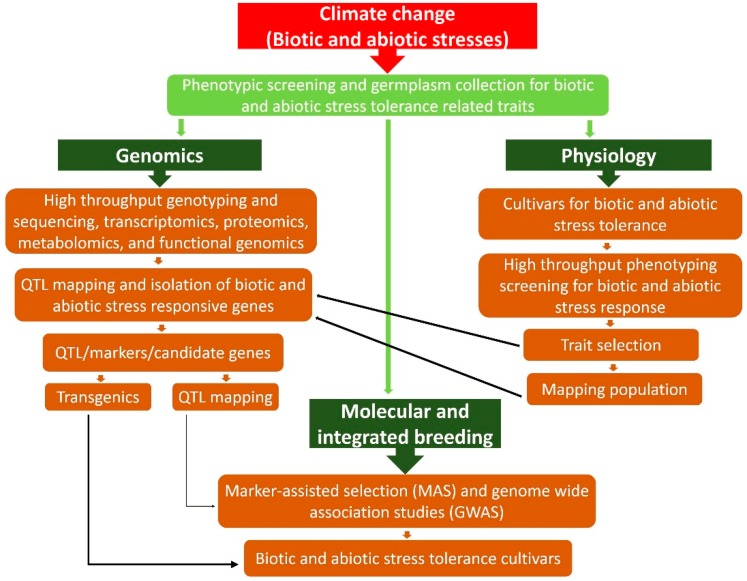
A step-wise presentation of physiological, molecular breeding and genomics approaches to develop biotic and abiotic stress tolerance cultivars.

Under various environmental stresses, plant breeding shows dynamic techniques in crop development and betterment. It gives a way to potentially guarantee food security and safety under harsh weather variations and help plants escape from various stresses through a crucial phase of plant growth by developing stress resistant cultivars [119]. Genetic divergence analysis is used for polymorphism, inbreeding, assessment, assortment, and recombination to attain plant perfection, and is amongst the main aspects for defining accomplished inbreeding. Genetic divergence analysis is considered a very important method for the development of new cultivars based on genetic distance and similarities [120,121]. For genetic studies landraces are a significant source, for example, a wheat landrace kept in data bank comprises broader genetic variance and is a valuable basis for stress resistance as it contains cultivars adjustable to diverse environmental stress [122]. Figure 6 demonstrates how molecular and integrated plant breeding are useful to develop the biotic and abiotic stress tolerance cultivars using genomics approaches like marker-assisted selection (MAS) and genome wide associated studies (GWAS).

### 7.3. Genetics and Genomics Strategies

#### 7.3.1. Omics-Led Breeding and Marker-Assisted Selection (MAS)

Omics approaches provide beneficial resources to elucidate biological functions of any genetic information for crop upgrading and development [123]. Different molecular markers are studied in population genomics across the environment in many individuals to find out novel variation patterns and help to find if the genes have functions in significant ecological traits [124]. In many crops, the breeding program is coupled with genomic approaches to achieve great heights in molecular breeding and to screen elite germplasms with multi-trait assembly [125] For the identification of phenotypes under different environmental variation associations, genetics and transcriptomic analysis are used [126] Genomics also enables investigation of the molecular mechanisms underlying the abiotic stress resistance. These approaches aid in the development of climate smart crops for better yield and production under different climate changes [127]. With the advent of high throughput sequencing and phenotyping, genomic-led breeding paved the way for identifying different stresses that are expected to adversely affect crop yield. Furthermore, the data available on environmental extremes, DNA fingerprinting, and quantitative trait loci (QTL) mapping allows the screening of elite germplasm under abiotic stresses [128]. QTL dissection of yield-related traits in crops under stress conditions permits the development of novel cultivars with better adaptability in abiotic stress [129]. Molecular plant breeding is an essential approach to enhancing crop yield and production in the presence of various biotic and abiotic stresses [130]. For speedy breeding progression marker-assisted selection (MAS) presents a crucial part in the betterment of crop traits and yield. With the advancement in crop genomics, DNA markers have been identified which are valuable for marker-assisted breeding [131]. 

The introduction of novel sequencing tools greatly eased the difficulty in researching genomic variants and lead towards the identification of huge amounts of DNA polymorphism, particularly single nucleotide polymorphism (SNPs) markers [132] Precision of QTL mapping enhanced on average from 10–30 centimogran to <1 cM [133,134] with the advancement of linkage maps [135]. The high-throughput phenomics approach is also contributing to increasing the accuracy of QTL mapping [136]. The association among genotypic and phenotypic data is crucial for enlightening the genetic basis of multiple traits [137]. By applying QTL mapping Haley and his colleagues successfully developed a wheat variety called “Ripper” which has the ability to withstand the drought conditions of Colorado, without affecting its grain yield and quality [138]. In 2009 Badu-Aparku and Yallou performed QTL mapping to screen elite maize germplasm with high yield under drought stress [139]. Merchuk-Ovant et al. (2016) conducted marker assisted selection studies on bread wheat (*Triticum aestivum* L.) and durum wheat (*Triticum turgidum* L.) to identify the QTLs related to drought stress [140]. Barley is cultivated on a wide range of land across the world but it is severely affected by drought stresses globally. QTL mapping of two novel barley cultivars that have been totally different in their response to drought stresses were selected and QTL mapping study performed for malting characters in double haploid. QTL investigation showed that MSA are specific reliable genomic sections regulating the malting feature which can be helpful [141]. Similarly, a recent QTL study was carried out to explore the epistatic mechanism and physiology of QTL for the elucidation of the targeted gene. Under drought stresses, 3 QTL were recognized such as qDTY6.2, qDTY6.1, and qDTY3.1, which have a considerable impact on grain productivity [142]. To be resistant under hyper temperature conditions three vital points on the genome of bread wheat have been predicted: 7D, 7B, and 2B [47]. Tahmasebi et al. (2016) performed QTL mapping for a recombinant inbred lines (RILs) population of wheat under different stress conditions of flooding, drought, heat, and a combination of both heat and drought simultaneously. QTL mapping showed a 19.6% variation in grain yield under these stress conditions. The authors concluded from this research that the molecular markers could be exploited to explore the unique allelic variations in wheat to enhance the potential to screen drought tolerant cultivars [143]. 

#### 7.3.2. Genome Wide Association Studies (GWAS) for Stress Tolerance

Genome wide association studies (GWAS) is a powerful tool for understanding the complete set of genetic variants in different crop cultivars to recognize allelic variant linked with any specific trait [144]. GWAS generally highlight linkage among SNPs and traits and based on GWAS design, genotyping tools, statistical models for examination, and results interpretation [145]. In many crops GWAS has been carried out to exploit the genetic process responsible for genetic resistance under climate change [146]. In plants, GWAS has widespread applications related to biotic and abiotic stresses. GWAS have been applied to describe drought tolerance [147], salt tolerance [148], and heat tolerance [149]. 

In *Arabidopsis thaliana* GWAS study was carried out by Verslues et al. (2013) aided by reverse genetic approaches to elucidate unique genes that accumulate proline under drought stress. The linkage among SNPs of both genotypic and phenotypic data were examined, and specific regions regulating proline accumulation were recognized. Similarly, different proteins controlling the pro-accumulation such as aMADS box protein, Universal Stress Protein A domain proteins, protein phosphatase 2A subunit A3, thioredoxins, ribosomal protein RPL24A, and mitochondrial protease LON1 were identified by using reverse genetics. This research gave insights for proline accumulation under drought stress conditions [150]. *Aegilops tauschii* is reported to have many resistance genes regulating the abiotic stresses [151]. A significant knowledge is required for the breeders to understand the genetic architecture of *Aegilops tauschii* to improve drought resilience. Qin et al. (2016) investigated 373 different varieties of *A. tauschii* to examine 13 traits controlling drought stress. For GWAS 7185 SNPs were designated to study the phenotypic behavior and carried out mixed linear model and general linear model to find the association between SNPs with phenotypic traits [152]. 

QTLs related to salinity resistance in plants were studied by using GWAS. Kumar et al. (2015) reported various genes regulating the salinity tolerance in rice by using infinium high-throughput SNPs arrays. Six thousand genotype-based SNP were constructed for genes related to stress and linkage among SNPs and phenotypic data were interpreted. QTLs for salt tolerance by genomic regions were mapped on chromosome numbers 1, 4, 6, and 7. A novel QTL present on chromosome number 1 was reported and was called “Saltol” which is associated with salt tolerance at seedling stage [153]. Lafarge et al. (2017) performed GWAS for genotyping 167 rice varieties for spikelet sterility (SPKST), and panicle micro-nutrient by applying 3 techniques of haplotype regression, single marker regression, and co-fitting of all markers to analyze the impact of heat during anthesis process. A significant association was present between SPKST, secondary traits, and 14 loci. These loci were investigated for functions related to heat shock proteins, controlling plant responses, development of gametophyte, cell division, and detecting abiotic stresses [149]. Chopra et al. (2017) reported various stress-tolerant genes in *Sorghum bicolor* associated with heat and cold stresses. GWAS was conducted for genotyping and phenotyping analysis. Thirty SNPs were identified for genes related to anthocyanin expression and carbohydrate metabolism, which are powerfully associated with cold stress at the seedling growth phase of sorghum. Similarly, 12 SNPs were discovered for heat stress at the seedling stage and controlled by the genes having functions in ion transport mechanism and sugar metabolism [154]. In another study Chen et al. (2017) examined *Sorghum bicolor* for heat tolerant traits such as leaf firing (LF) and leaf blotching (LB) at the vegetative phase of growth. To identify the association among SNPs with genotype and heat tolerance, GWAS was performed. Nine SNPs were closely linked with LF and five SNPs were identified for LB traits. Furthermore, 14 genes associated with SNPs were discovered that have stress-responsive expression to abiotic stresses [155].

#### 7.3.3. Genome Selection (GS) for Crop Improvement

Genomic selection (GS) is the exciting tool to revolutionize the crop improvement by using high-throughput phenotyping and marker densities to screen the elite germplasm, improving the polygenic traits and economical breeding line development [156]. Currently, the prospective of genomic selection (GS) to fast-track the speed of genetic achievements in main crops has stimulated the development of multi-environment designs for genomic estimation. Burgueño et al. (2012) proposed the first statistical design by applying a linear mixed model to G × E model [157]. Jarquín et al. (2014) suggested a system of modeling connections among an elevated dimensional combination of markers and environment that integrates with each other (G × E) [158]. Another model (GBLUP-type model) was proposed by Lopez-Cruz et al. (2015) in which regression of phenotypes was used for the interaction of marker × environment (M × E) [159]. The modern multi-environment model for genomic prediction was proposed by Cuevas et al. (2017) based on Bayesian model. These methods are applied on 4 wheat and 1 maize cultivars and CIMMYT data bank revealed that the G × E model have high significance rates and better genomic predictions as compared to other models [160]. 

Around 40 research studies based on GS have been published so far. Wheat is the most studied crop with 29 genomic selection studies. Moreover barley, oat, and durum wheat have 5, 2, and 1 research paper published. Diversity Array Technology (DArT) was the most promising maker used in GS followed by single nucleotide polymorphism (SNP) and genotyping by sequencing (GBS). These experiments showed that GS could be magnificently used in cereal breeding [161]. Genomic Selection (GS) designs were extensively developed for wheat to reveal the germplasms that have better ability to adapt in climate changes [162]. Crain et al. (2018) studied the different GS techniques to detect phenotypic data from high throughput phenotyping. At CIMMYT, heat and drought stresses were examined in 1000 elite wheat cultivars by using a high throughput phenomics approach [163].

#### 7.3.4. Genetic Engineered Plants for Stress Tolerance 

Biotechnology is an influential approach for genetic manipulation of the genome for the betterment of human beings. The genetic modification through biotechnology is a powerful strategy. Encouraging data is collected from genetics which can be exploited significantly to various biotic and abiotic stresses such as salinity, drought, heat, and cold. Identification of stress-responsive TFs are powerful findings to develop stress-resistant crop cultivars. These TFs can control the phenotypes of genes in genetic engineered crops associated with various stresses [164]. There are numerous transgenic plants which have been established by genetic engineering to tackle the biotic and abiotic stresses. These genetically engineered plants demonstrate significant resistance against climatic variations compared to normal plants [165,166].

Various transcriptions factor (TFs) are recognized as plant-specific TFs which includes AP2/ERFBP group [167]. This family of AP2/ERFBP TFs is responsible for many plant growth pathways and has functions in biotic and abiotic stress responses [168]. *AP2/EREBP* TFs are categorized into 4 sub-groups based on their similarity and numbers. The subfamilies consist of ERF TFs, DREB (dehydration-responsive element-binding protein), AP2 (Apetala 2), TFs, and RAV (related to *ABI3/VP1*). DREB and ERF are two major subfamilies which have been widely examined due to their role in plant biotic and abiotic responses [169]. The DREB TFs have significant regulating ability in various water deficit and cold stress conditions [170]. DREB TFs have been investigated in response to stresses in various plants species such as wheat, barley, maize, soybean, rice, tomato, and *Arabidopsis* [171,172,173]. In numerous experiments *DREB1* has been studied in rice and *Arabidopsis* for its controlling mechanism in cold stress while *DREB2* functions in drought, salinity, and high temperature stresses [174,175,176]. The *DREB1* TFs were over-expressed to develop transgenic *Arabidopsis* with a better ability to withstand salinity, drought, and freezing stresses [177,178]. Similarly, *DREB1* genes were introduced into the rapeseed, rice, tomato, and tobacco for cold stress resistance [179,180,181,182]. Many of the *DREB1* genes have also been purified from wheat, rye, maize, rice, and oilseed rape and have been transformed to develop transgenic crops against different abiotic stress [183,184]. Qin et al. (2007) isolated the *ZmDREB2A* gene from maize and over-expressed in *Arabidopsis* to developed transgenic *Arabidopsis* with improved resistance against drought stress [185]. Similarly Chen et al. (2007) revealed that the transgenic plants with over-expression of *GmDREB2* gene extracted from soybean showed significant tolerance against salt and drought resistance [186]. Mallikarjuna et al. (2011) successfully developed transgenic rice with the improved resistance against salinity and drought stresses by over-expressing the *OsDREB2A* gene [187].

The larger subfamily of AP2/EREBP TFs are ERF [188] and are responsible to regulate stress-tolerance genes in plants [189]. Under abiotic stresses these ERF genes are induced to hyper-express [190] results in the better tolerance against stresses in transgenic plants. Additionally, some ERF TFs are also involved both in biotic and abiotic stress tolerance due to their ability to regulate numerous hormonal biosynthesis pathways [191]. Zhu et al. (2014) studied that the introduction of *TaPIE1* in wheat has successfully improved the tolerance ability against chilling stress and resistance to pathogens [192]. Further study was carried out to develop transgenic tobacco by an over-expressed *GmERF3* gene. This transgenic tobacco has increased resistance against TMV, dehydration, and also toleration of salinity stress [193].

MYB TFs family called the myeloblastosis oncogene is a huge group of TFs discovered in eukaryotes and is extensively distributed in plants [194,195]. Various MYB TFs have been identified to regulate numerous biochemical and physiological pathways such as the cell cycle, hormonal biosynthesis, and primary and secondary metabolism. These TFs are also known to have functions in biotic and abiotic stress responses [196]. Li et al. (2015) have summarized various MYB TFs related to abiotic stress tolerance in plants and *Arabidopsis* [195]. Some of the MYB TFs such as *AtMYB61, AtMYB60,* and *AtMYB44* were identified to enhance the drought resistance in transgenic *Arabidopsis* by controlling the movement of stomata [197,198,199]. The *AtMYB96* gene was expressed in *Arabidopsis* as a regulator, either by ABA signaling pathways to impart drought resistance [200] or by controlling the biosynthesis process of cuticle wax [201]. Yang et al. (2012) developed transgenic rice by over-expressing the *OsMYB2* gene to improve the resistance of rice against chilling, salinity, and dehydration [202]. The *GmMYB76* gene isolated from soybean was successfully transformed into *Arabidopsis* for salinity and freeze resistance [203]. *MdMYB121* from apple was significantly transformed into apple and tomato to develop transgenic plants with enhanced drought and salt tolerance [204]. Chen et al. (2017) reported that the *ZmMYB30* gene isolated from maize was transformed into *Arabidopsis* to enhanced tolerance against salinity [205]. Similarly, the *MdSIMYB1* gene from apple was used to developed transgenic tobacco and apple with improved resistance against cold, drought, and salt stresses [206]. The *TaPIMP1* gene was expressed to develop transgenic wheat and it showed remarkable tolerance against drought and fungal pathogen *Bipolaris sorokiniana.* Hyper-expression of *TaPIMP1* was confirmed by microarray analysis [207]. In another experiment *TaPIMP1* TF was investigated for its regulating ability to tackle biotic and abiotic stresses in transgenic tobacco. Transgenic tobacco showed resistance against *Ralstonia solanacearum*, salinity, and drought stresses [208].

Another important family of TFs are WRKY which are extensively distributed in relation to abiotic [209] and biotic stresses in plants [210]. In transgenic plants WRKY genes were over-expressed to increase the abiotic stress tolerance such as in transgenic rice *OsWRKY11* gene was introduced to enhance its tolerance to heat and drought stresses [211]. Zhou et al. (2008) conducted an experiment on *Arabidopsis* to make it resistant against different stress conditions. *GmWRKY21* and *GmWRKY54* were over-expressed in transgenic *Arabidopsis* to improved resistance to cold and drought stress respectively [212]. Niu et al. (2012) investigated the *TaWRKY19* gene over-expression in transgenic wheat to freezing, drought, and salinity stresses [213]. Similarly, *TaWRKY1* and *TaWRKY33* isolated from wheat was used to develop transgenic *Arabidopsis* against drought and heat tolerance [214]. *ZmWRKY33* genes in maize have been induced for salinity, drought, freeze, and ABA stresses. Under salinity stress conditions transgenic *Arabidopsis* with over-expression of *ZmWRKY33* genes showed significant tolerance [215]. Dehydration tolerance was increased in transgenic Chrysanthemum with the over-expression of *CmWRKY1* [216].

NAC TFs have significant importance in many processes, such as flower growth, cell division, and stress-responsive regulation in the plant due to biotic and abiotic stresses [217,218]. Numerous NAC TFs have been discovered in a wide range of plants with sequenced genomes such as in rice with 151, in *Arabidopsis* 117 [219], in maize 152 [220], and in soybean 152 [221] NAC TFs have been identified. Additionally, a large number of NAC TFs have been reported to have direct association in abiotic stresses, such as in transgenic *Arabidopsis* 31 NAC genes, which have been identified for salinity tolerance [222], and in rice 40 NAC genes were identified against drought tolerance [223]. In sorghum the expression of *SbSNAC1* gene was induced by salt and drought stress and over-expression of this gene in transgenic *Arabidopsis* showed drought resistance [224]. Zheng et al. (2009) developed transgenic rice by over-expression of *OsNAC045* which conferred drought and salt tolerance [225]. Over-expression of *OsNAC1* in transgenic rice was studied for salinity and drought tolerance [226].

### 7.4. Genome Editing Strategies

Genome editing (GE) is the most powerful strategy to manipulate the plant genome by means of sequence-specific nucleases. GE for crop improvement has the remarkable ability to tackle food insecurity and develop a climate-smart agriculture system globally [227]. In traditional plant breeding approaches, genes are discovered to be associated with various important traits by means of mutation and conventional breeding strategies, which has recognized as a significant technique for the development of elite and high yielding germplasm [228]. Genetic diversity of various elite varieties has been substantially decreased due to the exploitation of important crops extensively that has, in various circumstances, been associated with the enhancement of the susceptibility to several abiotic and biotic stresses [229,230]. Plant breeding approaches have been greatly influenced by the GE tools and exploring new strategies for fast and accurate manipulations in crop genomes to protect them against different stresses and improve crop yield [231]. Thus, developing the novel modifications in the gene pool of various plant germplasm is required under abiotic and biotic stresses for the improvement of elite crop varieties with great ability to produce high yielding crops [232]. In genome editing technology site specific endonucleases are used comprising of zinc-finger nucleases (ZFNs), transcription activator like effector nucleases (TALENs), and CRISPR-Cas9 [233]. In contrast to the ZENs and TALENs genome editing tools, the CRISPR/Cas9 system is emerging as the most powerful GE strategy because it is economical, rapid, accurate, and enables multiple site specific editing within the genome [234]. 

#### CRISPR/Cas9 System for Crop Advancement

**Figure 7:**
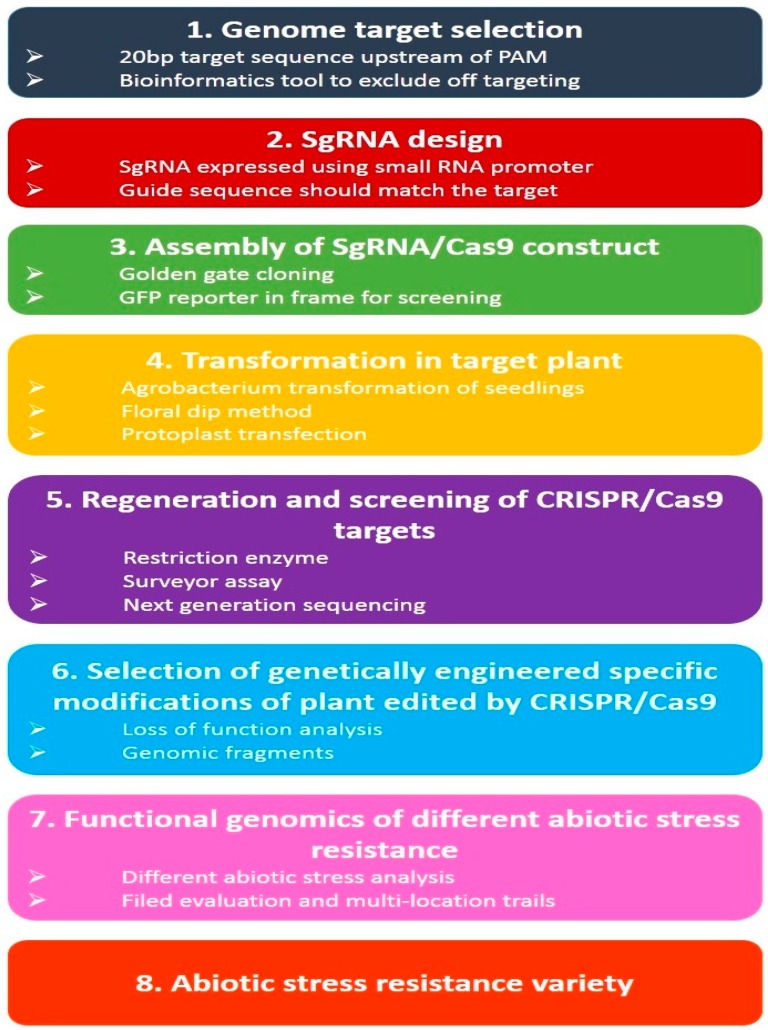
A model of CRISPR/Cas9 based genome engineering to develop transgenic plants or abiotic stress tolerance cultivars.

CRISPR/Cas9 is a modern genome editing strategy based on the prokaryotic defense mechanism triggered by type II RNA organization that offers protection to prokaryotes against attacking viruses [146,235,236]. Genome editing has been modernized by CRISPR-Cas9 assembly, by producing candidate gene mutants and knock down single nucleotides in a genome [237]. As compared to other genome editing tools like TALENs/ZFNs, CRISPR-based strategies have been tremendously exploited in plant genomes [238]. Moreover, it has great potential to aid crop breeding to establish high yielding and stress-resistant varieties [234]. Most significantly, the CRISPR/Cas9 tool is converting into a comprehensible environmentally friendly technique for the establishment of genome edited non-transgenic plants to tackle environmental extremes and guarantee food security [239]. A model of CRISPR/Cas9 based genome engineering to develop transgenic plants or abiotic stress tolerance cultivars is explained in Figure 7. 

CRISPR/Cas9 has been extensively carried out for plant genome editing to cope with abiotic and biotic stresses [240]. A study was conducted to disrupt the gene *TaERF3* and *TaDREB2* to produce abiotic stress resistance by using the CRISPR genome editing tool [241]. Similarly, 21 KUP genes have been identified in cassava which were hyper-expressed under abiotic stresses. Differential expression analysis of KUP genes revealed that they induced drought resistance [242]. For drought tolerance studies MAPKKK genes have been investigated by means of genome-wide analysis [243]. CRISPR/Cas9 was applied in rice for producing triplet mutants. Genes *TGW6, GW5,* and *GW2* have a function in regulating the seed size. By mutating this gene, the size of the seed was increased by 30% [244]. CRISPR technology has been adopted for a mutation in *Brassica napus* by knocking down the gene *CLVTA3* which resulted in more seed production. A similar strategy was applied to increase the wheat seed size by knocking down the *TaGW2* gene which has the ability to limit the seed size to increase [245]. Without any transformation of a gene, wheat has been developed with a low gluten level by using CRISPR/Cas9 technique [246]. Wang et al. (2017) studied the protein kinases 3 (*slmapk3*) gene to investigate its regulating mechanism to confer drought resistance in tomato. The CRISPR/Cas9 strategy was used to develop tomato mutant lines which showed a considerably enhanced concentration of proline, malondialdehyde, and H_2_O_2_. The tomato mutant lines were susceptible to more oxidative stress in drought. The outcomes of this study revealed the importance of the *SlMAPK3* gene in drought tolerance mechanisms and over-expression of this gene by genetic engineering provide improved drought tolerance [247]. The *SlAGL6* gene was knocked down by Klap et al. (2017) by using the CRISPR/Cas9 system to develop parthenocarpic fruits in response to heat stress. The resulting mutated tomato lines were the same as the normal plants having same shape and weight [248]. Shen et al. (2017) studied japonica rice by knocking out the *Osann3* gene. CRISPR/Cas9 technology was applied for mutant rice lines, resulting in the enhancement of tolerance under cold stress. This study showed the ability of the *OsANN3* gene in cold tolerance and that it could be a potential gene for transgenic rice with increased cold tolerance [249]. Herbicide tolerance was developed by knocking down *PmCDA1* gene in mutant rice lines with the help of CRISPR/Cas9 [250]. 

## 8. Conclusions

Climate changes are alarming the world by hampering agriculture and its products. Industrialization and poisonous gases cause global warming, which ultimately disturbs the world’s environment. Climate change has devastating effects on plant growth and yield. Abiotic stresses are the major type of stresses that plants suffer. To understand the plant responses under different abiotic conditions the most pressing current need is to explore the genetic basis underlying these mechanisms. Some bottleneck molecular and physiological challenges present in plants need to be resolved for better plant adaptation under abiotic conditions. Temperature fluctuations and variations in rainfall spells are a very crucial indicators of environmental stresses. Weather variations collectively have positive and negative outcomes but the negative effects are more thought-provoking. It is very difficult to overcome the imbalance in agriculture by climate change. How to tackle this problem and what strategies we should apply are still ambiguous. Hence, researchers need to focus on optimizing plant growth and development in abiotic stresses. For crop resistance against biotic and abiotic stresses, propagating novel cultural methods, implementing various cropping schemes, and different conventional and non-conventional approaches will be adopted to save agriculture in the future. Breeding approaches will help to develop climate resilient crops with better adaptability under drought and heat. Genome wide association studies (GWAS), genomic selection (GS) with high throughput phenotyping, and genotyping strategies are significant in identifying the different genes for crop improvement under climate change. Genetic engineering approaches have been significantly applied to develop transgenic plants with enhanced resistance against different biotic and abiotic stress responses. In future, we have to make eco-friendly genome edited crops through a CRISPR/Cas9 mediated genome editing to battle against climate change.
